# Owren's Disease: A Rare Deficiency

**DOI:** 10.7759/cureus.17047

**Published:** 2021-08-10

**Authors:** Madiha Ehtisham, Muhammad A Shafiq, Muhammad Shafique, Hassan Mumtaz, Muhammad Naveed Shahzad

**Affiliations:** 1 Medicine, Rawalpindi Medical University, Rawalpindi, PAK; 2 Internal Medicine, California Institute of Behavorial Neurosciences and Psychology, Fairfield, USA; 3 Internal Medicine, Rawalpindi Medical University, Rawalpindi, PAK; 4 Internal Medicine, Holy Family Hospital, Rawalpindi, PAK; 5 Clinical Research, Shifa International Hospital Islamabad, Islamabad, PAK; 6 Critical Care Medicine (COVID ICU) and Surgery (Urology/Orthopedics), Kahuta Research Laboratories Hospital, Islamabad, PAK; 7 Pediatrics, Holy Family Hospital, Rawalpindi, PAK; 8 General Medicine, Surrey Docks Health Centre, London, GBR; 9 Urology, Guy's and St Thomas' Hospital, London, GBR

**Keywords:** haematology, clotting factors, leiden, congenital disorders, blood coagulation disorder

## Abstract

Factor V deficiency is a rare bleeding disorder, which may be due to acquired inhibitors or biallelic mutations. Factor V deficiency due to homozygous or compound heterozygous mutation (also known as Owren's disease or parahemophilia) has an estimated prevalence of one in one million people.

A 22-year-old female was admitted for evaluation of longstanding menorrhagia. Anatomic abnormalities were excluded, and prolonged prothrombin time (PT) and partial thromboplastin time (PTT) were identified. Mixing studies followed by specific factor assays and genetic testing enable identification of factor V deficiency, for which fresh frozen plasma (FFP) or factor V concentrates are therapeutic.

Specific clotting factor assay followed by mixing studies and genetic studies is essential for the diagnosis of congenital factor V deficiency. Deranged PT and activated partial thromboplastin time (APTT) with normal factor I level must be evaluated for the disorder of clotting factors and must be managed by FFP administration or plasma-derived factor V concentrate wherever available.

## Introduction

Factor V deficiency is a rare bleeding disorder, which may be due to acquired inhibitors or biallelic mutations. Factor 5 deficiency due to homozygous or compound heterozygous mutation (also known as Owren's disease or parahemophilia) has an estimated prevalence of one in one million people [1]. Deficiencies in coagulation factors may present with bleeding of any severity from minor ecchymoses to life-threatening hemorrhage. Factor V deficiency is rare and may be genetic or acquired due to an inhibitor [2].

Acquired factor V deficiency from an inhibitor, evident when coagulation parameters fail to correct during mixing studies, has been associated with multiple triggers including drugs, malignancy, and infections. Genetic deficiency is more likely with onset in youth. Patients typically (40%) present with mucosal bleeding including epistaxis and menorrhagia [3].

## Case presentation

The patient is a 22-year-old female who is married for two months, has no children, and is a resident of Attock, Pakistan. She was admitted to the gynecology unit of a tertiary care hospital with a history of heavy menstrual periods. She was meticulously examined and evaluated by the attending physicians in the gynecology department, and no evidence of any gynecological abnormalities was found. She was enquired about the systemic bleeding disorder. Interestingly, the patient had a history of easy bruising and purpura on minor trauma since childhood. On examination, there was pallor, multiple petechiae, purpura throughout the body. For the next step of management, her clotting profile was ordered. She had a deranged clotting profile, i.e., prolonged prothrombin time (PT) and activated partial thromboplastin time (APTT) but normal bleeding time (Tables 1, 2). Her platelets count was also normal.

**Table 1:** PT and APTT time observed on different days of admission PT, Prothrombin time; APTT, activated partial thromboplastin time.

PT (sec) (10 to 20 sec)	APTT (sec) (24 to 39 sec)	Day of admission
39.65	77.87	2nd
50	>120	13th
40	77	15th
19 (patient + adsorbed plasma)	32 (patient + control)	15th
45 (patient + aged serum)		15th

**Table 2:** Clotting factors

Clotting factors	Value in the patient	Normal range
VIII	143%	50% to 149%
IX	116%	50% to 163%
One/Fibrinogen	260 mg/dl	180 to 350 mg/dl

Our primary differential diagnosis was clotting factor disorder. Her clotting factors VIII and IX were normal that ruled out hemophilia A and B, respectively. Ristocetin assay test for functional platelet disorder also came back normal that ruled out Von Willebrand disease. Factor I was normally given in the disseminated intravascular coagulation (DIC) profile. As both PT and APTT were deranged, we reached a conclusion to investigate the levels of clotting factor involved in the common pathway namely factors II, IV, and X (Table 3).

**Table 3:** Lab investigations ELISA, enzyme-linked immunosorbent assay; FDP, fibrin degradation product.

Bleeding time	04 min 42 seconds	Normal
Platelet count	396 10^9^/L (2^nd^ DOA); 364 10^9^/L (15^th ^DOA)	150 to 400 10^9^/L
Ristocetin cofactor activity	197% (Normal)	50% to 200.9%
Hess time	Negative	
Hepatitis B serology via ELISA	< 0.245 (Negative)	
Hepatitis C serology via ELISA	< 0.245 (Negative)	
FDPs/D-dimers	<150 ng/Ml (Normal)	< 250 ng/Ml normal value

Factor 5 deficiency occurs in liver disease as well as in DIC. The patient's test results for hepatitis B and hepatitis C virus were negative. DIC was also ruled out as her D-dimers, fibrinogen, and platelet count were normal. We then proceeded toward the idea of factor 5 deficiency as the DIC, and the most common liver diseases are ruled out. So most likely, it is a genetic factor 5 deficiency, which was going on for a while in this patient and exacerbated once she got married. After her marriage, she complained of heavy menstrual bleed and landed in the gynae emergency room, where her PT APTT levels were measured and factor V deficiency was diagnosed.

Her menorrhagia was treated with fresh frozen plasma (FFP) transfusion. On discharge, she was given iron supplements and folate for anemia prophylaxis. The patient was advised to seek medical aid in case of bleeding. She was counseled that in case of moderate to severe bleed, she may need FFP transfusions.

## Discussion

Bleeding disorders present with a spectrum of signs and symptoms may be found incidentally for the workup of systemic bleeding episodes such as hematemesis, melena, hemoptysis, easy bruising, ecchymosis, or menorrhagia [4]. This case presented with abnormal heavy menstrual bleeds, and she was referred to the gynecology department for abnormalities of the gynecological tract. Cases of hematemesis, melena, and menorrhagia must be evaluated for systemic bleeding disorders investigating the organ system where the bleeding occurs. The attending physician must envision bleeding disorders even when the patient is at the risk of bleeding due to pathology of the organ system where hemorrhage takes place; for example, if a patient with a history of chronic liver disease comes with upper gastrointestinal bleed, systemic bleeding disorders must be kept in mind while making differential diagnoses. It is an important differential diagnosis along with variceal bleed in a patient with decompensated chronic liver disease [5].

We conducted mixing studies to differentiate between factor deficiency and factor V inhibitor. PT and APTT were successfully corrected by mixing studies, which negated the presence of any antibody against clotting factor V. Mixing studies are done to determine whether a PT or partial thromboplastin time (PTT) is elevated due to a factor deficiency or a factor inhibitor (antibodies to specific factors). It is done by mixing the patient's plasma with control plasma. If the mixed plasma PT and PTT normalize, the PT and PTT prolongation is due to a factor deficiency. If they do not normalize, the prolongation is due to a factor inhibitor [6].

Hemophilia A, Von Willebrand disease, and vitamin K deficiency are relatively common bleeding disorders [7]. While other bleeding disorders like factor V deficiency, Bernard-Soulier syndrome, and Glanzmann thrombasthenia are rarely seen clinically, genetic disorders like hemophilia usually present congenitally or in early childhood [8]. While other disorders may present at varying ages, this patient presented with menorrhagia in adulthood, but her childhood petechiae and easy bruising hint toward a possible congenital factor V deficiency. In cases of congenital factor V, the patient may be completely asymptomatic or there may be petechial hemorrhages and ecchymosis.

Congenital factor V deficiency causing uncontrolled bleeding is seldom seen [9]. However, the triggering factors that lead to noticeable spontaneous bleeding are still unknown. In this case, the patient had menarche at the age of 12, and she had normal menstrual bleeds till the age of 22 when she developed menorrhagia. We were unable to identify any triggering factors that cause menorrhagia in this patient. In bleeding disorders, wounding and trauma are the most common triggers for heavy bleeding. Minor surgical procedures in infants such as circumcision and injection may result in heavy bleeding, raising the suspicion of congenital bleeding disorders like hemophilia [10]. Family history is extremely important in genetic bleeding disorders such as hemophilia A. But in this case, the patient denied similar bleeding complaints or ecchymosis in any other family member. This suggests that factor V deficiency is probably a non-inheritable disorder, but this observation must be reinforced by further scientific evidence and epidemiological studies.

Acquired factor V deficiency has also been reported, and it is mediated by acquired factor V inhibitors. Certain drugs such as Cephradine and Cefepime, members of the cephalosporin family, cause acquired factor V deficiency [11-13]. Other causes of acquired factor V deficiency are usually urinary tract infection, hemodialysis, myeloma, and amyloidosis [14]. The mutation that leads to congenital factor V deficiency is yet unidentified. Some studies suggest that a spectrum of mutations including missense, nonsense, and frameshift may be present in the F5 gene [15], and it is also unknown if factor V deficiency is familial. We recommend genetic analysis of these patients to identify genetic mutation leading to factor V deficiency.

## Conclusions

Factor V deficiency is a rare disorder. These cases may present with bleeding with a history of petechial hemorrhages, non-traumatic bruises, and ecchymosis. Bleeding disorders must be considered whenever there is a history of such suspicious bleeding. Specific clotting factor assay followed by mixing studies is essential for the diagnosis. These cases are managed by FFP administration although the plasma-derived factor V concentrate is also available. Genetic studies should be done in patients with congenital factor V deficiency.
